# The Week's Work

**Published:** 1909-12-11

**Authors:** 


### The Week's Work.

### INSURANCE FEES AND THE HOSPITAL STAFF.

An interesting case has recently occurred in a New York hospital, where a member of the staff refused to sign a death certificate of a policy-holder who had died in the hospital. The representative of the insurance company tendered the usual fee, but the superintendent of the institution forbade its acceptance, on the ground that all fees paid belong to the hospital, as such, and not to individual members of the staff. That being the case, the medical man refused to sign the certificate. In commenting on the case the local Press strongly condemned the action of the member of the staff, and urged the general adoption by other hospitals of the rule the superintendent of this institution has so far enforced. In England no medical man is allowed to charge for a death certificate. He must sign the first certificate, certifying death, free of charge, and this must be forwarded to the registrar. He is, however, allowed to issue duplicate certificates, and to make whatever charge he likes for these; and it not infrequently happens that the insurance authorities, or the deceased's friends, request the issue of such a duplicate. In hospital practice here, we believe, it is invariably the rule that such fees belong to the member of the staff who signs the duplicate certificate, just as coroners' inquest fees, reports in accident cases, and other stray moneys that come into the hands of the underpaid resident, are, by courtesy, left in his hands. The principle on which the American rule relies, however, is recognised by law in this country, where no resident can claim a fee for attending an inquest upon a patient who has died in hospital.

### THE PUBLIC POINT OF VIEW.

The objection raised by the American Press that if hospital residents were allowed to refuse duplicate certificates unless they were paid for them, is hardly a reasonable one in this country. In general practice, we believe, the practitioner nearly always refuses to give a duplicate, simply because it is a point of courtesy to refer all applicants to the registrar, whose prerogative it is to issue duplicate certificates at a certain fixed charge. There is no hardship to the public, therefore, in the case of a hospital resident charging two guineas for such a duplicate; all the patient's friends have to do is to go to the registrar and get as many duplicates as they want at a much lower price, without troubling the certifying resident in the least. It would be well, therefore, for hospital residents to adopt the custom which obtains in private practice, and look upon the issue of such duplicates as the prerogative of the registrar, and make a rule to refuse all requests, pointing out to the applicants where and how they may obtain their certificates. The main point is that *the* death certificate—the only one that has any legal value—must be signed free of charge, and must be filed with the registrar on the usual printed form. All other certificates granted by the certifying medical man are merely formal notifications of the patient's death, whereas the duplicates issued by the registrar have all the weight of an officially stamped and authorised document.

### THE HOSPITAL CAT.

In most, if not in all, hospitals there are to be found one or more cats, serving not only for ornament to the ward and for the relaxation of convalescent patients, but honestly earning their living by helping to rid the wards of mice. In colonial hospitals, where the rodent plague is far greater than in English institutions, the necessity for these ward cats is imperative, and their care is entrusted to the nurses or wardmaids, while if they are found to be growing too old or too fat for active service they are promptly superannuated and replaced by younger and more active felines. At Hong-Kong, we believe, there is a special regulation which provides for the maintenance of several cats in the wards, in conformity with the official requirements that every large house should possess at least three cats. In the German colony of Togo, where an up-to-date little hospital is to be found, it has recently been discovered that no cats are available for hospital purposes, and the Governor has accordingly ordered half-a-dozen to be sent to the hospital with his compliments. In his official decree he points out that it is highly desirable to keep the houses as free as possible from rats and mice, which are known as plague carriers, in view of the vicinity of the Gold Coast where plague is endemic. Koch has already praised the cat very highly as a help in the eradication of plague, and the Governor of Togo has by his general order put the hospital variety upon an exalted pedestal, from which only the total extinction of that disease can remove it.

### OBJECTIONS TO THE WARD CAT.

In European hospitals many objections have been raised to the free perambulations of the cat. An early hospital writer, in one of his reports, notes the fact that in a certain hospital in Holland cats were allowed to "live in the wards, lie on the patients' beds, and generally to conduct themselves as if they were in ordinary family houses." That objection, however, cannot be alleged against the cats in a properly conducted modern hospital, where the cat is usually an unobtrusive, mild, and gentle animal, whose presence in the children's wards is a source of constant and continual enjoyment to the little patients. Such attached cats are kept scrupulously clean; they enjoy a daily bath, and rarely leave the precincts of the ward or wander outside into the corridors, and certainly do not so far forget their dignity as to make elevated excursions upon the roof. At the same time it is only fair to state that no one has yet achieved the task of properly disinfecting a cat, and that there are valid objections to allowing the ward cat to stray from a fever ward into a convalescent ward, or to be at large at all in a room where infectious cases are housed. Its proper duty is to keep the administrative and economic portion of the ward clear of mice and rats, and its visits to the general ward should be confined to the day room. With these restrictions there cannot be any great objection to the hospital cat.

### ROYAL PORTSMOUTH HOSPITAL FINANCE.

At the meeting of the Committee of Management of this Hospital, on November 19, dealing with the financial difficulties, Dr. Claremont said he approved the proposal for reducing the period during which tickets were available to one month. He is further reported to have said that, as the cost of out-patients' treatment was more than covered by the money which the tickets represented, tickets for the out-patients should remain available for the original time. We are glad to see that those responsible for hospital finance have awakened to the recommendations on this subject which we have so often urged in these columns. Tickets of admission, which were originally devised as an inducement, to use no stronger word, for subscriptions, have proved themselves to be, as at present issued, an expensive luxury, and the point made by Dr. Claremont is well worth noticing. If it is the general rule that the cost of treatment in the out-patient department is more than covered by the money which the tickets bring in, a useful hint is given to the body of hospital treasurers to expand this source of income at the same time as they contract the losses which tickets for the in-patients' department have slowly and steadily accumulated.

### THE WARD CONCERT.

Equally interesting are our Dublin patient's remarks on ward entertainments, and they are particularly so at this season, when active preparations are being made for the approaching Christmas festivities in nearly all of our hospitals. We have on more than one occasion been struck by the super-excellence of some of these artistic entertainments offered to hospital patients, and have wondered in what degree they have been appreciated. Perhaps our Dublin patient is right when he says that Beethoven and Mendelssohn are not the best musical fare for the average institutional inmate, and that popular and more homely programmes would be infinitely better appreciated than a strictly classical one. He records an impression of a fine classical concert in a Dublin ward with the amusing comments of his neighbours on what "thim fine looking young wummen wur singin'," and how much the majority of patients were grieved that "Johnny Hart" did not figure on the programme. A Christmas entertainment in a hospital has always its sad side. The screened bed serves as a skeleton at the feast, and inevitably detracts from a full enjoyment of the fun. For that reason it is imperative that the programme for such entertainments should be arranged not, as is very often the case, at haphazard, but with care and discrimination. Carelessly arranged programmes, however good the performers are, cannot be expected to meet with general approval, and in such entertainments the wishes and tastes of the majority of patients should have due weight. Some of the best ward concerts we have attended were arranged by the ward staff, acting in accordance with the expressed or implied wishes of the patients, and aided by loyal and willing outside talent, which is always at the disposal of our institutions that know how and where to apply for it.

### MANCHESTER ROYAL INFIRMARY.

At the last meeting of the Board of Management of the Manchester Royal Infirmary on Tuesday, November 30, it was decided to establish a department for the bedside investigation of cancer. A good deal of excellent work in this direction has been carried on during the past few months by individual effort on the part of members of the medical staff and others, and recently donations amounting to £150 have been placed in the hands of one of the members of the honorary staff for the purpose of promoting cancer research. This has been considered an excellent opportunity of bringing the matter formally before the Board of Management, with the result that it has been decided to take the question up. Arrangements will at once be made to fit up a temporary special laboratory for three or four workers. The Board has announced its willingness to receive special donations towards the expenses of the department, and there is every prospect of further developments. The investigations will be purely clinical. Manchester has just erected its new Royal Infirmary, which will eventually accommodate 600 patients. It has a large and increasing medical school with excellent laboratories and teaching accommodation, and this further step into the field of research indicates the determination of the hospital authorities that the institution shall be in the forefront of all branches of medical and surgical science.

### THE DRUG MARKET.

At the recent public sale of drugs held in Mincing Lane, the demand was surprisingly quiet, seeing that six weeks will elapse before tne next auction. The feature of the sale was gum ammoniacum, which fetched extreme prices, but in the case of the other drugs which were sold there was no such marked rise or fall in value as would effect an alteration in the prices of galenical preparations made from them. The Japanese monopoly has announced a small advance in the price of camphor, and although there seems no reason to expect any pronounced increase in the value of refined camphor, the present is not an unfavourable time to make purchases to cover the requirements of the next two or three months. Seidlitz powder has been slightly reduced in price. The demand for cod-liver oil has fallen off slightly, but a spell of cold weather would undoubtedly increase the consumption of this drug, and prices would probably tend higher. Potassium iodide, so far as present indications show, is not likely to be reduced in price in the near future, and purchasers would probably not gain by delaying their orders. As regards rectified spirit and tinctures, no advantage would be gained by making purchases to cover more than present needs.

